# Transparency and Accountability of Medicaid Section 1115 Waiver Demonstration Programs Under the Affordable Care Act

**DOI:** 10.1001/jamanetworkopen.2020.22035

**Published:** 2020-10-26

**Authors:** Leo Lopez, David Silvestri, Joseph S. Ross

**Affiliations:** 1 National Clinician Scholars Program, Department of Internal Medicine, Yale School of Medicine, Yale University, New Haven, Connecticut; 2 NYC Health + Hospitals, New York, New York; 3 Section of General Internal Medicine, Department of Internal Medicine, Yale School of Medicine, Yale University, New Haven, Connecticut; 4 Department of Health Policy and Management, Yale School of Public Health, New Haven, Connecticut; 5 Center for Outcomes Research and Evaluation, Yale–New Haven Health System, New Haven, Connecticut

## Abstract

This cross-sectional study examines US states’ compliance with both Patient Protection and Affordable Care Act demonstration program annual reporting and Medicaid guidelines for Section 1115 experimental program evaluations.

## Introduction

Under Section 1115 of the Social Security Act, the Secretary of Health and Human Services may waive Medicaid requirements and permit states to pilot experimental models of health care delivery. In 2015, these programs accounted for more than $100 billion in federal expenditures. To date, 43 states have secured 55 active waivers to test various Medicaid program changes, including work requirements, benefit restrictions, or managed long-term services and support systems.^1,2,3^ To promote transparency and accountability of these publicly funded “demonstration” programs, the Patient Protection and Affordable Care Act (ACA) mandated in 2012 that states receiving waivers publish both annual progress reports and periodic program evaluations.^4^ In 2014, the Centers for Medicare & Medicaid Services (CMS) provided explicit guidance on the expected content of these evaluation reports and established an office to monitor their quality in 2015.^3^ Our objective was to examine states’ compliance with both ACA demonstration program annual reporting and CMS guidelines for program evaluations.

## Methods

Using publicly available CMS administrative records, we conducted a 2-part cross-sectional study of state Medicaid 1115 waiver demonstration programs. In the first part, we used CMS administrative records to identify all demonstration programs active during 2011-2013 and 2016-2018. These years correspond to before ACA reporting requirements and after CMS reporting guidance, respectively. In any given year, we excluded programs in their first year of approval because insufficient time had elapsed for required reporting. Among remaining programs, we identified the presence or absence of a publicly available annual report in each year by searching the CMS.gov website, on which the agency is required by law to publish annual reports and evaluations.^4^ If none was identified, we also searched state Medicaid websites. We determined the overall percentage of program-years with publicly available annual reports for both 2011-2013 and 2016-2018, and compared percentages with χ^2^ testing.

The second part focused on the 2016-2018 publicly available annual reports. We determined a report to be a program evaluation according to whether it was identified among the CMS administrative records for a respective state as an “evaluation.” By reviewing each evaluation, we determined the presence or absence of the following report components outlined in the CMS guidance^5^: executive summary, background, hypothesis, methods, results, conclusions, interpretations and policy implications, and lessons learned and recommendations. For each component, we used descriptive statistics to summarize the total percentage of evaluation reports with each component present. This study follows the Strengthening the Reporting of Observational Studies in Epidemiology (STROBE) reporting guideline reporting guideline for cross-sectional studies.

## Results

From 2011-2013, we identified 45 active demonstration programs across 32 states. Based on years of program activity, a maximum of 126 annual reports from these programs would be expected; however, only 6 (4.8%) were publicly available. From 2016-2018, we identified 36 active demonstration programs across 24 states. Based on years of program activity, a maximum of 93 annual reports from these programs would be expected; 54 annual reports (58%) were publicly available (*P* < .001) (Table).

**Table.** Medicaid Section 1115 Waiver Programs: Availability of Annual Reports and Presence of Reporting Components for Program Evaluations

	No. (%)	
	2011-2013	2016-2018
Demonstration programs	45	36
States	32	24
Publicly available annual reports identified/expected	6/126 (4.8)	54/93 (58)
Program evaluations		20
Executive summary		10 (50)
Background		15 (75)
Hypothesis		13 (65)
Methods		14 (70)
Results		18 (90)
Conclusions		9 (45)
Interpretations and policy implications		4 (20)
Lessons learned and recommendations		4 (20)

From among publicly available annual reports between 2016 and 2018, we identified 20 that were program evaluations, representing 36 demonstration programs (24 states). The median number of components reported per program evaluation was 5 (interquartile range, 2-6). The most commonly reported program evaluation components were results (n = 18; 90%) and general background information (n = 15; 75%), whereas interpretations and policy implications and lessons learned and recommendations were least commonly reported (n = 4 for both; 20%). None of the 11 evaluations that were submitted to CMS for renewal reported interpretations and policy implications.

## Discussion

Despite an increase in the public availability of annual reports for Section 1115 demonstration programs after the ACA and CMS’s evaluation guidance, more than 40% were not publicly available. Moreover, when demonstration program evaluations were made publicly available, they consistently failed to report key evaluation components.

There are limitations to consider, including our inability to account for special terms and conditions negotiated between CMS and individual states that may dictate requirements for reporting frequency. In addition, we did not contact federal or state officials when annual reports or evaluations were not identified. However, given the importance of Medicaid Section 1115 waivers to pilot innovative models of health care delivery, and the amount of federal funding involved, enforcement of existing CMS rules to ensure accountability is needed, particularly for program renewals. Availability of program reports and evaluations allows the broader public to better understand the effect of these programs on Medicaid beneficiaries.
